# Extracellular Vesicles Derived from *Elaeocarpus braceanus* Alleviate DSS-Induced Ulcerative Colitis in Mice Through Multiple Pathways

**DOI:** 10.3390/nano16150968

**Published:** 2026-08-06

**Authors:** Wen-Bo Feng, Tong Liu, Mu-Yao Liu, Hui-Ying Fu, Lu Li, Zheng-Yi Zhou, Qiang Cai, Yu-Xin Chen

**Affiliations:** 1 Hubei Key Laboratory of Industry Microbiology, Key Laboratory of Fermentation Engineering (Ministry of Education), School of Life and Health Sciences, Hubei University of Technology, Wuhan 430068, China; 2 State Key Laboratory of Hybrid Rice, Hubei Hongshan Laboratory, College of Life Sciences, Wuhan University, Wuhan 430072, China

**Keywords:** ulcerative colitis, *Elaeocarpus braceanus*, extracellular vesicles, intestinal barrier, TLR4/MyD88/NF-κB, intestinal flora

## Abstract

Aim of the study: This study aims to isolate extracellular vesicles derived from *Elaeocarpus braceanus* fruits (EBDEVs) and evaluate their alleviating efficacy as nature nanoparticles against dextran sulfate sodium (DSS)-induced ulcerative colitis (UC). Methods: EBDEVs were isolated by differential and density gradient ultracentrifugation, then characterized for morphology, size, stability, and composition. Their anti-inflammatory activity was assessed in LPS-stimulated RAW264.7 macrophages. In vivo, acute UC was induced in C57BL/6 mice by 2.5% DSS. Disease severity, intestinal barrier integrity, TLR4/MyD88/NF-κB pathway activation, and gut microbiota composition were evaluated. Results: EBDEVs exhibited a typical spherical structure and were rich in bioactive components such as lipids, flavonoids, and terpenoids. Macrophages readily internalized them and significantly inhibited LPS-induced NO production. In UC mice, EBDEVs ameliorated weight loss, colon shortening, and tissue damage, while reducing serum inflammatory cytokines. EBDEVs restored intestinal barrier function by regulating tight junction proteins. Mechanistically, EBDEVs suppressed the activation of TLR4/MyD88/NF-κB and downstream NLRP3 inflammasome inflammatory signaling cascades, and remodeled the dysregulated gut microbiota structure. Conclusions: EBDEVs alleviate DSS-induced UC in mice by repairing the intestinal barrier, inhibiting inflammatory pathways, and modulating gut microbiota.

## 1. Introduction

Ulcerative colitis (UC), a major subtype of inflammatory bowel disease, features chronic, relapsing, and diffuse inflammation of the colonic mucosa that usually initiates in the rectum and spreads continuously along the colon [1,2,3]. Typical clinical manifestations of UC comprise bloody diarrhea, abdominal pain, vomiting, weight loss, and hematochezia [4,5]. Its high recurrence and refractory characteristics severely impair patients’ well-being and quality of life [6], and more importantly, markedly elevate the risk of colorectal cancer [7]. Global incidence of UC has been increasing, with a higher prevalence in Europe and North America, especially among young adults [8].

The pathogenesis of UC involves complex crosstalk among genetic factors, intestinal flora dysbiosis, mucosal immune overactivation, epithelial barrier defects, and environmental triggers, leading to a protracted, recurrent, and incurable disease course [9,10]. Current mainstream therapies for UC comprise aminosalicylates, corticosteroids, immunomodulators, and biological agents [11,12]. Yet, these strategies are limited by poor cost-effectiveness, high drug resistance rates, and adverse effects (e.g., infection, malignancy, dermatitis) [13,14]. Thus, given the limitations of traditional therapies, exploring novel, cost-effective treatment strategies with lower toxicity is of importance for human health.

Extracellular vesicles (EVs), recognized as key mediators of intercellular communication, hold significant promise in disease treatment and drug delivery [15,16]. Among these, plant-derived extracellular vesicles (PDEVs) have emerged as a cutting-edge research hotspot in nanomedicine and drug delivery systems. Owing to their natural nanoscale structure, excellent biocompatibility, low immunogenicity, gastrointestinal stability after oral administration, and inherent cross-kingdom biological regulatory capability, PDEVs are regarded as promising novel natural nanomedicine agents for the treatment of intestinal inflammatory diseases [17,18]. They can efficiently penetrate the intestinal microenvironment, be internalized by intestinal epithelial cells and immune cells [19], and concurrently exert anti-inflammatory, intestinal barrier-protective and microecological regulatory effects, showing great potential for the development of oral targeted nanomedicine against UC [20].

*Elaeocarpus braceanus* (*Elaeocarpus braceanus* Watt ex C. B. Clarke) is an evergreen broad-leaved tree, endemic to regions such as Yunnan and Tibet in China [21]. Its fruit possesses a unique flavor profile, described as sour and astringent with a lingering sweetness, and is processed into products like preserved fruit and juice. Also, as a medicinal and edible plant resource with a long application history, its fruit exhibits properties such as clearing heat and detoxifying, stopping bleeding and diarrhea [22,23]. Furthermore, studies have revealed that its fruit contains various primary and secondary metabolites, including alkaloids, amino acids, nucleotides, flavonoids, coumarins, lipids, phenolic acids, and tannins [23]. Its extracts have demonstrated multiple pharmacological effects, such as antioxidant, antihyperglycemic, and anti-inflammatory activities [24,25,26]. To date, no study has systematically explored the therapeutic value of its fruit extracellular vesicles as a novel oral nanotherapeutic for UC.

In this study, we isolated extracellular vesicles derived from *Elaeocarpus braceanus* fruits (EBDEVs), characterized their morphology and composition, assessed their in vitro anti-inflammatory activity, and further investigated their protective effects and potential mechanism of UC in vivo.

## 2. Materials and Methods

### 2.1. Reagents

*Elaeocarpus braceanus* fruits were obtained from Yunnan Province and authenticated by Dr. Gao Zhou at the Institute of Traditional Chinese Medicine Health Industry, China Academy of Chinese Medical Sciences (Nanchang, China). The Bradford Protein Quantification Kit (R21252) and DiO dye (S26181) were supplied by Yuanye Biotechnology Co., Ltd. (Shanghai, China). DSS was produced by MP Biomedicals (molecular weight: 36,000–50,000, Solon, OH, USA), and 5-aminosalicylic acid (5-ASA) was provided by Yien Chemical Technology Co., Ltd. (Shanghai, China). Murine serum concentrations of TNF-α, IL-1β, TFN-γ and IL-6 were analyzed using ELISA kits sourced from Solebao Technology Corporation (Beijing, China). The HiScript^®^ III 1st Strand cDNA Synthesis Kit (+gDNA wiper) (R312-02) and ChamQ^®^ Universal SYBR qPCR Master Mix(Q711-03) were supplied by Vazyme Biotech, Ltd. (Nanjing, China).

### 2.2. Isolation and Purification of EBDEVs

Fresh *Elaeocarpus braceanus* fruits were washed, pitted, and homogenized with PBS (material-to-liquid ratio 1:6). The juice was filtered and sequentially centrifuged at 4 °C (1000× *g* for 10 min, 4000× *g* for 20 min, 10,000× *g* for 30 min) to remove debris. The supernatant was collected and ultracentrifuged (BECKMAN/Optima XE-90 Ultracentrifuge, Beckman Coulter Inc. Pasadena, CA, USA) at 120,000× *g* for 1 h at 4 °C, and the resulting pellet was resuspended in PBS. Further purification was performed via sucrose density gradient centrifugation (8%, 30%, 45%, 60%, *w*/*v*) at 150,000× *g* for 2 h. The 30–45% sucrose band was collected, diluted, and ultracentrifuged again at 120,000× *g* for 1 h. The final pellet was resuspended in PBS, quantified by the Bradford method, and stored at −80 °C.

### 2.3. Characterization of EBDEVs

The morphology of EBDEVs was observed using transmission electron microscopy (TEM, JEM-1400 Plus, JEOL, Akishima, Japan). The particle size and zeta potential were measured using a nano laser particle size analyzer (Zetasizer Nano ZS90, Malvern Panalytical, Malvern, UK), and the particle concentration was determined using nanoparticle tracking analysis (NTA, Zetaview-PMX120-Z, Particle Metrix GmbH, Inning am Ammersee, Germany).

### 2.4. Gastrointestinal Stability of EBDEVs

The gastrointestinal stability of EBDEVs was assessed by measuring the changes in particle size of EBDEVs in simulated gastric fluid (SGF, pH = 1.2) and simulated intestinal fluid (SIF, pH = 6.8). The formulations of simulated gastric fluid and simulated intestinal fluid are listed in Supplementary Table S1.

### 2.5. Component Analysis of EBDEVs

We obtained the EBDEVs by the method described in Section 2.2, divided them into 3 equal portions of 200 µL each, and sent them to the Zhongke e-Test team (Wuhan, China) for Metabolomics analysis.

### 2.6. Cell Culture

Mouse mononuclear macrophages RAW264.7 (No. GDC0143) were obtained from the China Center for Type Culture Collection (Wuhan University, Wuhan, China). Cells were cultured in RPMI 1640 medium supplemented with 10% fetal bovine serum and 1% penicillin–streptomycin solution at 37 °C in a 5% CO_2_ incubator.

### 2.7. Cell Viability Determination

RAW264.7 cells were seeded at 5 × 10^3^ cells/well and incubated with graded concentrations of EBDEVs at 1.56, 3.13, 6.25, 12.5, 25, 50, 100 and 200 µg/mL for 24 h. Cell viability was then determined using the Cell Counting Kit-8 (CCK-8) colorimetric assay according to the manufacturer’s instructions.

### 2.8. Cell Uptake of EBDEVs

EBDEVs were labeled with DiO dye at 37 °C for 30 min, ultracentrifuged to remove free dye, and filtered through a 0.22 µm membrane. DiO-labeled EBDEVs were incubated with RAW264.7 cells, and cellular uptake was observed under fluorescence microscopy (BZ-X800E, KEYENCE Corporation, Osaka, Japan).

### 2.9. NO Production and Detection

Nitric oxide (NO) production was detected by the Griess reagent method. RAW264.7 cells were seeded at 5 × 10^5^ cells/mL, pretreated with EBDEVs (25, 50 µg total protein/mL) for 1 h, then stimulated with 1 µg/mL lipopolysaccharide (LPS) for 24 h. Supernatant was mixed with Griess reagent, and absorbance was measured at 540 nm.

### 2.10. Animal Experiment Design

Thirty male C57BL/6 mice (6–8 weeks old, 20 ± 5 g) were purchased from Wuhan Luobin Life Science Technology Co., Ltd. (License No.: SYXK(E)2024-0149, Wuhan, China). After adaptive feeding, mice were randomly divided into 5 groups (n = 6): control, DSS model, 5-ASA (200 mg/kg), EBDEV-L (5 mg total protein/kg body weight), and EBDEV-H (15 mg total protein/kg body weight). 2.5% DSS induced acute UC in drinking water for 6 days. Corresponding drugs were administered daily by gavage for 7 days. On day 8, mice were euthanized under carbon dioxide anesthesia, and blood, colon tissues, and colonic contents were collected for subsequent analysis. All animal procedures were approved by the Animal Care and Use Committee of Hubei University of Technology (HBUTLL20230033).

### 2.11. DAI Score

During the experiment, body weight, stool consistency, and fecal occult blood (assessed by the o-toluidine method) were monitored. The Disease Activity Index (DAI) score was calculated as the average of weight loss, stool consistency, and occult blood scores according to the criteria in Table S2.

### 2.12. Histopathological Analysis and Mucus Staining of the Colon

Fixed colon tissues were dehydrated, embedded, and sectioned (5 µm thickness). Colon morphology was assessed by Hematoxylin and Eosin (H&E) staining, Alcian Blue (AB) staining, and Periodic Acid-Schiff (PAS) staining. Stained sections were observed under a light microscope. Histopathological scores were assigned according to the criteria in Table S3.

### 2.13. Assay of Inflammatory Cytokines in Mouse Serum

Serum samples stored at −80 °C were thawed. The levels of TNF-α, IL-1β, IFN-γ, and IL-6 in mouse serum were measured using ELISA kits according to the manufacturers’ protocols.

### 2.14. Immunohistochemistry

Tissue sections were boiled in citrate buffer for antigen retrieval, blocked with 3% H_2_O_2_ and 3% BSA, then incubated with primary antibodies overnight at 4 °C and secondary antibodies for 1 h. Staining was visualized by DAB and hematoxylin and observed under light microscopy.

### 2.15. Real-Time PCR

Quantitative real-time PCR (qPCR) (CFX96 Touch, Bio-Rad Laboratories, Inc., Hercules, CA, USA) was performed to detect the mRNA expression levels of relevant proteins in colon tissue. Primer sequences are listed in Table S4. Total RNA was extracted from colon tissues using TRIzol reagent under low-temperature homogenization, and the concentration was measured by Nanodrop 2000 (Thermo Fisher Scientific, Wilmington, DE, USA). RNA was reverse-transcribed into cDNA using a cDNA synthesis kit. qPCR was performed using AceQ Universal SYBR qPCR Master Mix. The 2^−ΔΔCT^ method was used to determine relative gene expression levels normalized to the control group, with β-actin as the internal reference gene.

### 2.16. Western Blot

Western blotting was performed to detect the expression of related proteins in colon tissue. Colon tissues from each group were homogenized in lysis buffer and centrifuged at 12,000× *g* for 10 min to obtain supernatants. Total protein concentration was determined using the Bradford Protein Quantification Kit. Equal amounts of protein (20 µg per sample) were separated by 10% SDS-PAGE and transferred onto PVDF membranes. Membranes were blocked with 5% non-fat milk at room temperature for 30 min. Membranes were incubated with primary antibodies overnight at 4 °C, then incubated with secondary antibodies for 1 h. Protein bands were detected using an electrochemiluminescence (ECL) system. The gray values of the bands were quantified using ImageJ software (ImageJ, 1.53a), and the relative expression of each protein was calculated as the ratio of the target protein to the internal reference (β-actin).

### 2.17. Gut Microbiota Analysis

Gut microbiota analysis was performed on the intestinal feces collected from mice in the control group, model group, and EBDEV-H group. Microbial DNA was extracted from fecal samples, and the V3–V4 region of the 16S rRNA gene was amplified, and primer sequences are listed below: 338F (5′-ACTCCTACGGGAGGCAGCAG-3′), 806R (5′-GGACTACHVGGGTWTCTAAT-3′). Purified PCR products were sequenced, and effective tags were clustered into OTUs (97% similarity). Sample richness and diversity, differences at the phylum and genus levels, principal component analysis (PCA), and LEfSe analysis among groups were analyzed using the platform provided by BGI (Wuhan, China), which performed the entire sequencing service.

### 2.18. Statistical Analyses

GraphPad Prism 8 software was used for plotting. SPSS 25 statistical software was used for one-way analysis of variance (ANOVA) and difference significance (Duncan multiple comparisons). Differences between groups were considered statistically significant at *p* < 0.05, *p* < 0.01, and *p* < 0.001.

## 3. Results

### 3.1. Isolation and Identification of EBDEVs

**Figure 1:**
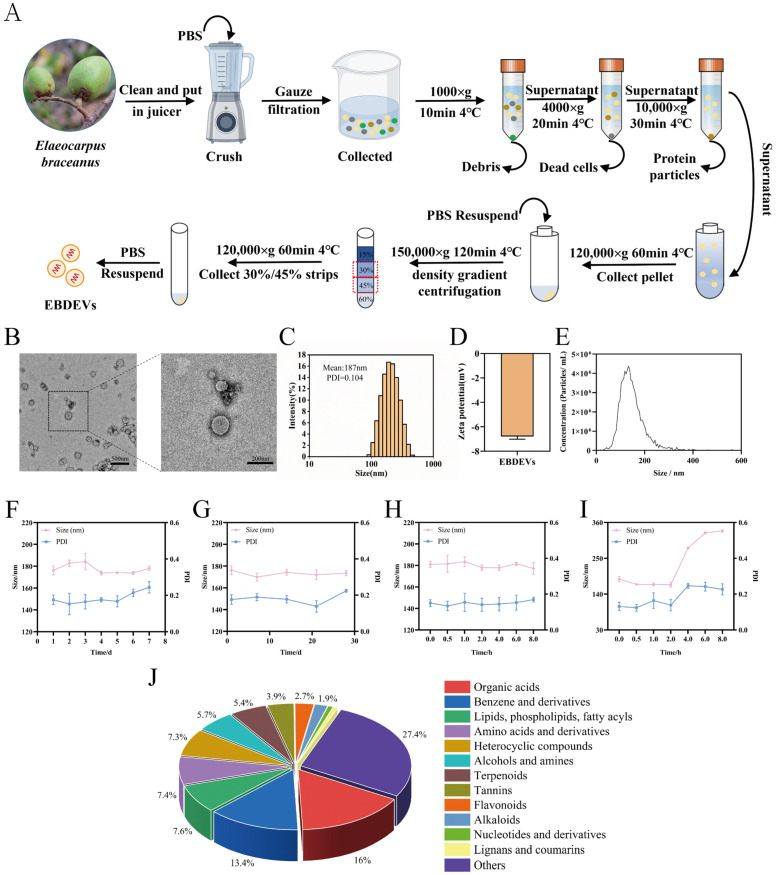
Characterization of EBDEVs. (**A**) Protocol for the isolation and extraction of EBDEVs. (**B**) Transmission electron microscopy image of EBDEVs. (**C**,**D**) EBDEV size distribution and zeta potential. (**E**) NTA particle concentration and size distribution analysis. (**F**) Changes in particle size and PDI of EBDEVs at 4 °C; data are expressed as mean ± SD, n = 3. (**G**) Changes in particle size and PDI of EBDEVs at −80 °C; data are expressed as mean ± SD, n = 3. (**H**) Changes in particle size and PDI of EBDEVs in SGF; data are expressed as mean ± SD, n = 3. (**I**) Changes in particle size and PDI of EBDEVs in SIF; data are expressed as mean ± SD, n = 3. (**J**) Metabolomics identified the classes of compounds in the EBDEVs.

EBDEVs were successfully isolated from *Elaeocarpus braceanus* fruit juice by differential centrifugation and sucrose density gradient ultracentrifugation (Figure 1A). TEM observation revealed a typical spherical morphology (Figure 1B), with an average hydrodynamic particle size of 187 nm and a PDI of 0.104 (Figure 1C). The zeta potential was −6.79 ± 0.22 mV (Figure 1D). NTA showed the majority of EBDEVs were 135 ± 1.4 nm in diameter and a particle concentration of 8.1 × 10^11^ particles/mL (Figure 1E). EBDEVs remained stable for 1 week at 4 °C and 1 month at −80 °C (Figure 1F,G). The particle size and PDI of EBDEVs remained stable in SGF. After 2 h of incubation in SIF, both the particle size and PDI of EBDEVs increased, whereas the vesicles still retained favorable nanoscale size and dispersibility (Figure 1H,I). Metabolomics analysis confirmed that EBDEVs were rich in lipids, amino acids, and organic acids (Figure 1J), as well as diverse natural products including flavonoids, terpenoids, tannins, and alkaloids (Table S5).

### 3.2. In Vitro Cell Experiments Using EBDEVs

**Figure 2:**
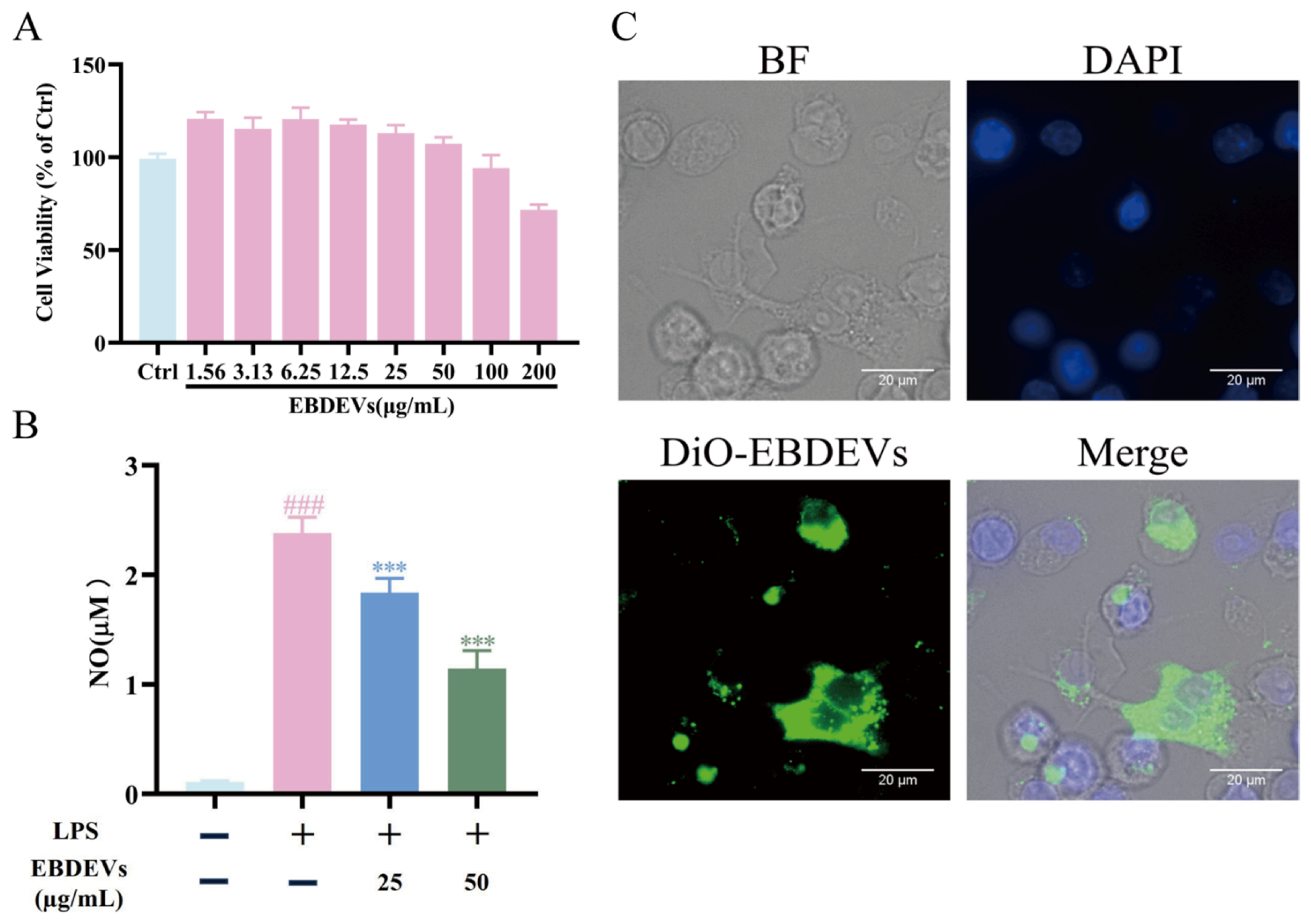
In vitro uptake and anti-inflammatory experiments with EBDEVs. (**A**) Effect of EBDEVs on cell viability of RAW 264.7. (**B**) The effects of EBDEVs on NO release from RAW 264.7 cells. (**C**) Images of RAW264.7 uptaking EBDEVs. Data are expressed as mean ± SD, n = 4. ### *p* < 0.001 versus the ctrl group; *** *p* < 0.001 versus the LPS group.

EBDEVs showed no cytotoxicity to RAW264.7 cells at 100 μg/mL. Calculations indicate that the IC_50_ value is 1001 μg/mL (Figure 2A). Within the range of EBDEVs concentrations that did not reduce cell viability (Figure 2A), 25 µg total protein/mL and 50 µg total protein/mL were selected as the low and high doses for further in vitro experiments. Both doses suppressed LPS-induced NO production in macrophages (Figure 2B). An in vitro uptake assay verified efficient uptake of DiO-labeled EBDEVs by RAW264.7 cells (Figure 2C).

### 3.3. Therapeutic Efficacy of EBDEVs in DSS-Induced UC Mice

**Figure 3:**
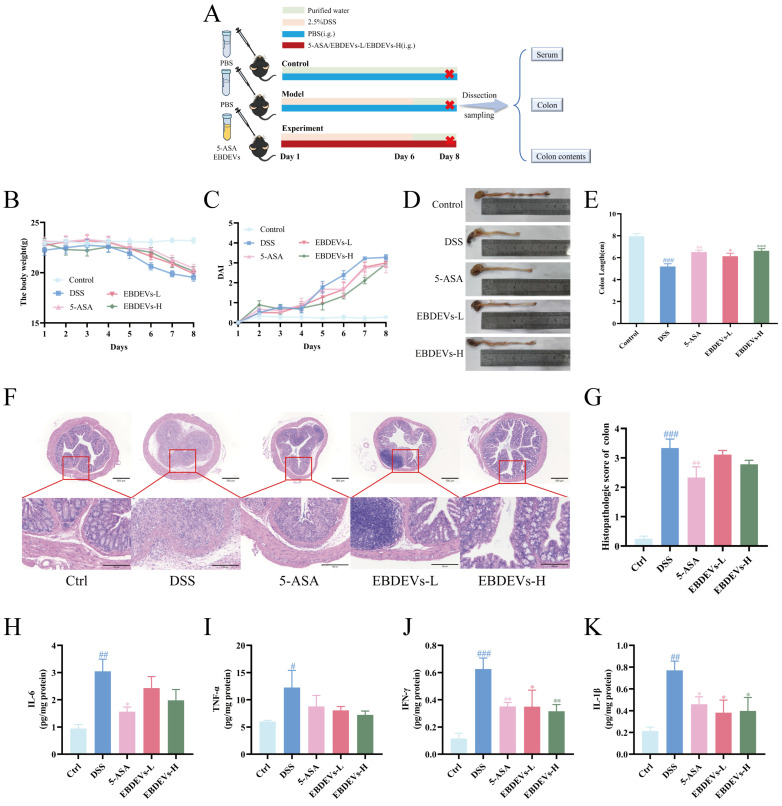
EBDEVs alleviate DSS-induced UC. (**A**) Schematic diagram of the modeling and dosing regimen of the DSS-induced UC model in C57BL/6 mice. (**B**) Body weight; data are expressed as mean ± SE, n = 6. (**C**) Disease activity index score; data are expressed as mean ± SE, n = 6. (**D**,**E**) Colon length comparison; data are expressed as mean ± SE, n = 6. (**F**,**G**) Histopathology of colonic tissues by H&E staining and histological score; data are expressed as mean ±SE, n = 6. (**H**–**K**) Protein levels of serum IL-6, TNF-α, IFN-γ, and IL-1β determined by ELISA; data are expressed as mean ± SE, n = 3. # *p* < 0.05, ## *p* < 0.01, ### *p* < 0.001 versus the ctrl group; * *p* < 0.05, ** *p* < 0.01, *** *p* < 0.001 versus the DSS group.

Mice in the DSS group presented lethargy, severe diarrhea, and hematochezia, accompanied by progressive body weight loss and an elevated DAI score. In contrast, treatment with 5-ASA and EBDEVs improved the physical activity of mice, with milder body weight loss and a lower DAI score (Figure 3B,C). The DSS group showed a significant shortening in colon length. Treatment with 5ASA and EBDEVs significantly alleviated this shortening (Figure 3D,E). H&E staining revealed that the control group had neatly arranged colonic crypts, intact goblet cells, and no inflammatory infiltration. Conversely, the DSS group displayed severe mucosal inflammatory infiltration, crypt destruction, and massive goblet cell loss. Treatment with 5-ASA and EBDEVs markedly alleviated DSS-induced colonic pathological damage. Histopathological scoring showed that the score of the 5-ASA group was lower than that of the EBDEV groups and had a significant difference compared with the DSS group (Figure 3F,G).

Serum ELISA results revealed that compared with the control group, IL-6 (Figure 3H), TNF-α (Figure 3I), IFN-γ (Figure 3J), and IL-1β (Figure 3K) levels were significantly elevated in the serum of DSS-treated mice. All treatment groups reduced these pro-inflammatory cytokine levels. The levels of IFN-γ and IL-1β in the three treatment groups exhibited a significant decrease compared to the DSS group, and IL-6 levels in the 5-ASA group showed a significant decrease relative to the DSS group. However, while the TNF- α levels in the three treatment groups were decreased compared to the DSS group, the decrease was not statistically significant.

### 3.4. Effects of EBDEVs on the Intestinal Barrier of UC Mice

**Figure 4:**
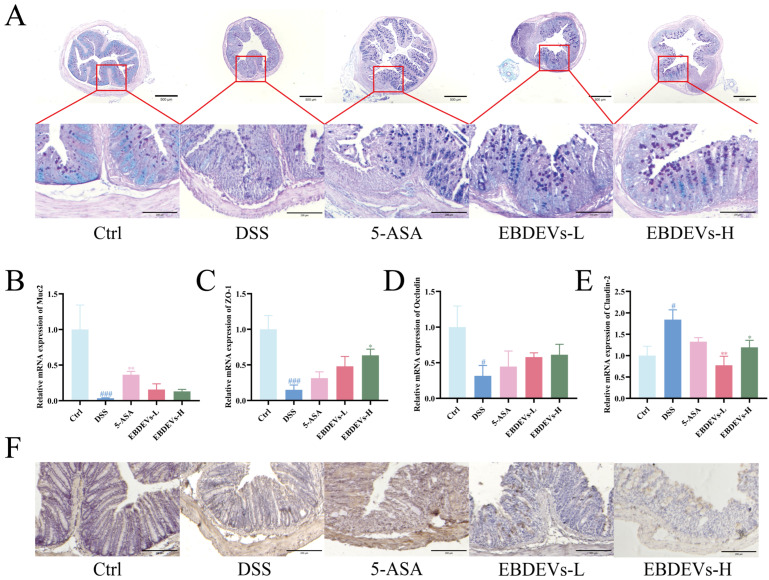
The effect of EBDEVs on the colonic mucous layer and tight junctions in UC mice. (**A**) The effect of EBDEVs on colonic goblet cells and the mucous layer in UC mice (AB-PAS staining). (**B**–**E**) The effect of EBDEVs on the mRNA levels of Muc2, ZO-1, Occludin, and Claudin-2 in UC mice. Data are expressed as mean ± SE, n = 4–6. (**F**) The protein expression level of ZO-1 was detected by immunohistochemistry. # *p* < 0.05, ### *p* < 0.001 versus the ctrl group; * *p* < 0.05, ** *p* < 0.01, versus the DSS group.

In Figure 4A, blue color indicates goblet cells, and purple color indicates mucin. The results showed that the control group had abundant goblet cells and sufficient mucin content in the mucosal layer. Mice in the DSS group exhibited severe damage to colonic structure, a reduction in the number of goblet cells, and a decrease in mucin content. Treatment with 5-ASA and EBDEVs inhibited these changes, resulting in an increased number of goblet cells in the mouse colon and a slight recovery of mucin content (Figure 4A). After administration of EBDEVs, the mRNA expression level of Muc2, which was significantly decreased by DSS, increased slightly but without significance. In contrast, 5-ASA treatment showed a limited but significant increase (Figure 4B). In the DSS group, the mRNA expression levels of ZO-1 and Occludin were significantly lower than those of the control group. In contrast, the mRNA expression level of Claudin-2 was significantly higher than that of the control group. After administration of EBDEVs, the mRNA expression levels of both ZO-1 (Figure 4C) and Occludin (Figure 4D) in the colon tissues of mice increased, and the mRNA expression level of Claudin-2 (Figure 4E) significantly decreased, with the effect of EBDEV being stronger than that of 5-ASA. In Figure 4F, the brown DAB staining signal indicates the ZO-1 protein; the results showed that ZO-1 content was reduced in the DSS group compared with the control group. EBDEV treatment notably increased ZO-1 protein expression in mouse colonic tissues (Figure 4F).

### 3.5. Effects of EBDEVs on the TLR4/MyD88/NF-κB Signaling Pathway

**Figure 5:**
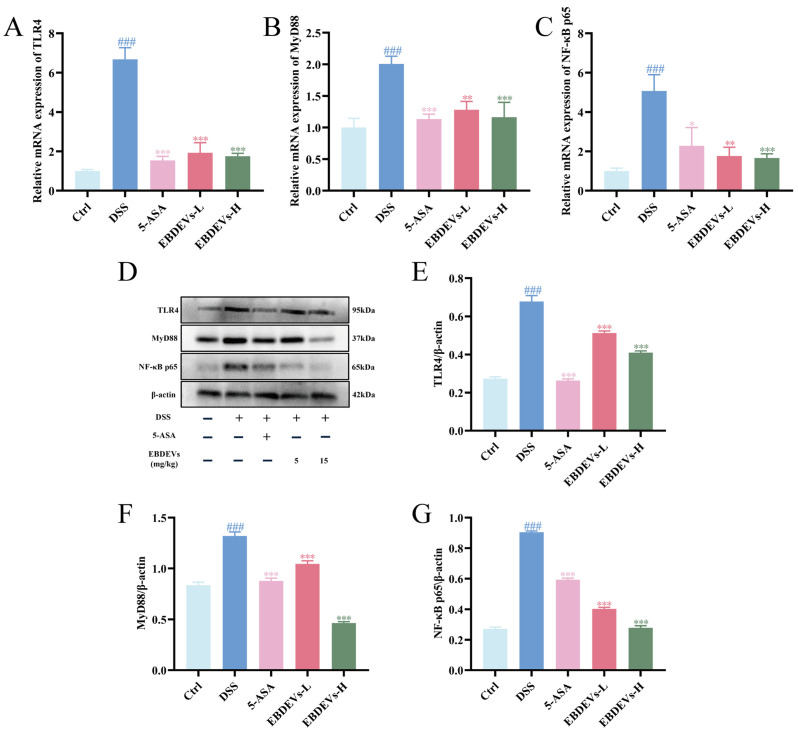
EBDEVs inhibit the TLR4/MyD88/NF-κB signaling pathway in vivo. (**A**–**C**) Effect of EBDEVs on the mRNA levels of TLR4, MyD88, and NF-κB p65 in UC mice; data are expressed as mean ± SE, n = 4–6. (**D**) The protein expression of TLR4, MyD88, and NF-κB p65 was determined by Western blotting. (**E**–**G**) Quantification of TLR4, MyD88, and NF-κB p65 protein; data are expressed as mean ± SE, n = 3. ### *p* < 0.001 versus the ctrl group; * *p* < 0.05, ** *p* < 0.01, *** *p* < 0.001 versus the DSS group.

The qPCR results showed that the mRNA expression of TLR4 (Figure 5A), MyD88 (Figure 5B), and NF-κB p65 (Figure 5C) was significantly increased after DSS administration. Treatment with 5-ASA and the different doses of EBDEVs reversed the increased expression of these genes to varying degrees. Western blot (Figure 5D) results showed that DSS significantly increased the protein expression levels of TLR4 (Figure 5E), MyD88 (Figure 5F), and NF-κB p65 (Figure 5G) in mouse colon tissue. Compared with the DSS group, EBDEV administration significantly reduced the expression levels of these three proteins.

**Figure 6:**
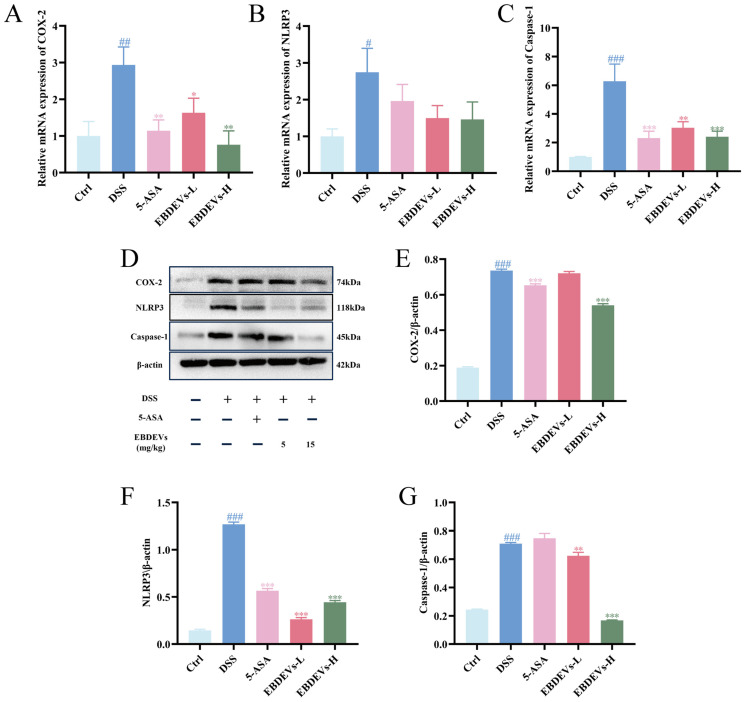
EBDEVs inhibit the mRNA and protein expression of COX-2/NLRP3/Caspase-1 in vivo. (**A**–**C**) Effect of EBDEVs on the mRNA levels of COX-2, NLRP3, and Caspase-1 in UC mice; data are expressed as mean ± SE, n = 4–6. (**D**) The protein expression of COX-2, NLRP3, and Caspase-1 was determined by Western blotting. (**E**–**G**) Quantification of COX-2, NLRP3, and Caspase-1 protein; data are expressed as mean ± SE, n = 3. # *p* < 0.05, ## *p* < 0.01, ### *p* < 0.001 versus the ctrl group; * *p* < 0.05, ** *p* < 0.01, *** *p* < 0.001 versus the DSS group.

Real-time PCR experiments revealed that the mRNA expressions of COX-2 (Figure 6A), NLRP3 (Figure 6B), and Caspase-1 (Figure 6C) significantly increased after DSS administration. After administration of EBDEVs, the expression levels of the above genes decreased. Among the two EBDEV administration groups, the mRNA expression levels of COX-2 and Caspase-1 showed significant differences compared to the DSS group. Meanwhile, Western blot (Figure 6D) results showed that DSS administration significantly increased the expression levels of COX-2 (Figure 6E), NLRP3 (Figure 6F), and Caspase-1 (Figure 6G) proteins in mouse colon tissues. After treatment with EBDEVs, the expression levels of these three proteins decreased, with NLRP3 and Caspase-1 showing significant differences compared to the DSS group.

### 3.6. Effects of EBDEVs on the Gut Microbiota of UC Mice

**Figure 7:**
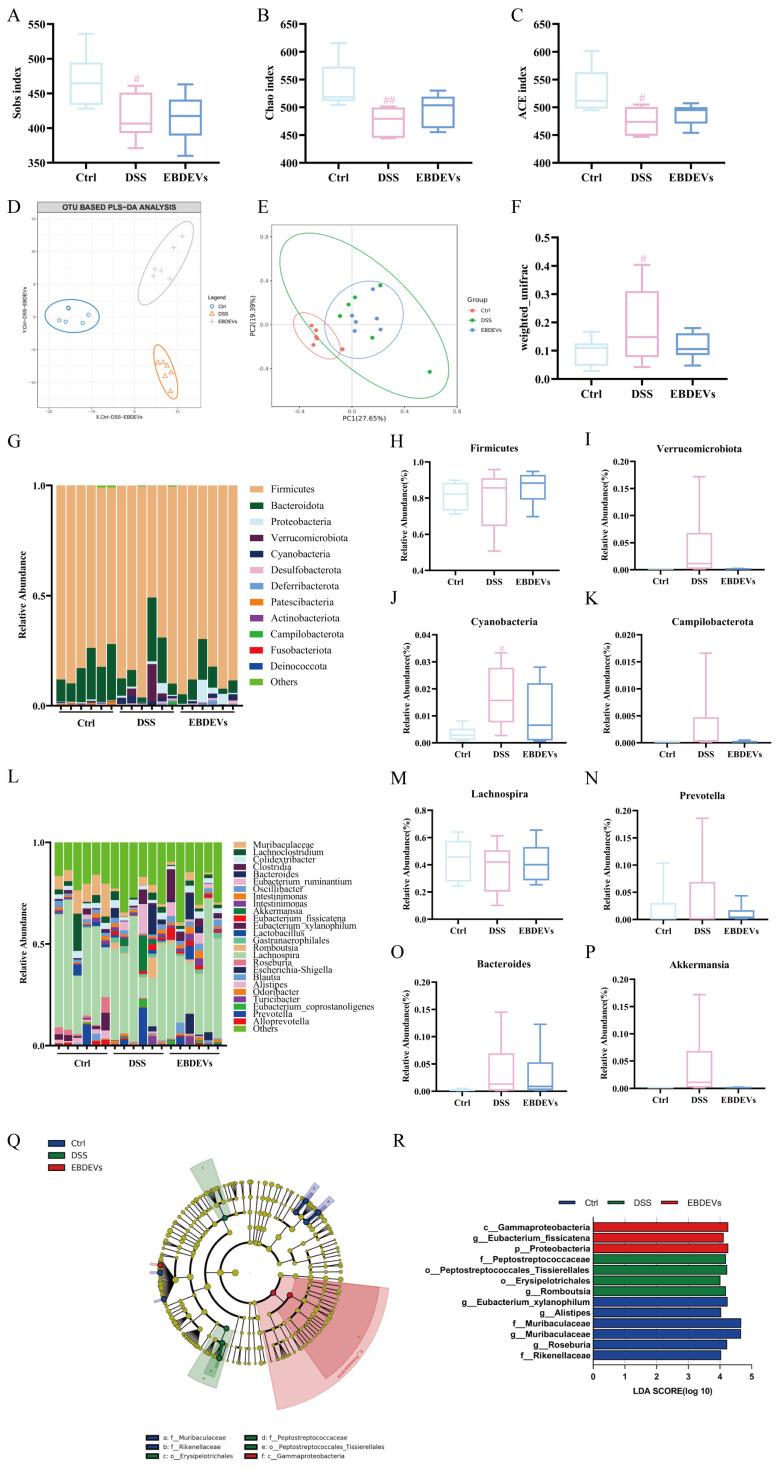
Effects of EBDEVs on the gut microbiota of UC Mice. (**A**–**C**) Alpha diversity index. (**D**) PLS-DA analysis. (**E**) PCA. (**F**) Beta diversity analysis. (**G**–**K**) Relative abundance of colonic microorganisms in mice at the phylum level. (**L**–**P**) Relative abundance of colonic microflora in mice at the genus level. Data are expressed as median ± Q, n = 6. (**Q**) Cladogram based on OTU; circles from the innermost to outermost layers represent the taxonomic levels of phylum, class, order, family, and genus, respectively. Each small circular node represents a taxon, and the node diameter is proportional to the mean relative abundance of that taxon. Yellow nodes indicate no significant difference among groups; blue nodes indicate taxa significantly enriched in the control group; green nodes indicate taxa significantly enriched in the DSS group; red nodes indicate taxa significantly enriched in the EBDEV group. (**R**) Histogram of LDA value distribution (LDA > 4). # *p* < 0.05, ## *p* < 0.01 versus the ctrl group.

The Sobs index represents the number of observed OTUs (Figure 7A), while the Chao and ACE indices reflect the richness of the microbial community (Figure 7B,C). The results showed that, compared with the control group, the Sobs, Chao, and ACE indices were significantly decreased in the DSS group, while these indices were elevated after EBDEV treatment. PLS-DA analysis based on OTUs indicated differences in gut microbial composition between different groups (Figure 7D). Principal component analysis and beta diversity analysis (based on weighted UniFrac distances) revealed greater variability in species composition in the DSS group. In contrast, the control and EBDEV groups showed less variation (Figure 7E,F).

Phylum-level compositional analysis showed that *Firmicutes* was the most dominant phylum in all three groups, accounting for nearly 80% of the mouse gut microbiota (Figure 7G). Evaluation of the relative abundance of *Firmicutes* (Figure 7H), *Verrucomicrobiota* (Figure 7I), *Cyanobacteria* (Figure 7J), and *Campilobacterota* (Figure 7K) indicated that the phylum composition of the EBDEV group was closer to that of the control group relative to the DSS group. At the genus level, *Lachnospira* was the most abundant genus, with relative abundances of 44%, 37%, and 41% in the control, DSS, and EBDEV groups (Figure 7L,M). Compared with the control group, the DSS group showed increased proportions of *Prevotella* (Figure 7N), *Bacteroides* (Figure 7O), and *Akkermansia* (Figure 7P). In contrast, these genera in the EBDEVs group returned to levels similar to the control group.

LEfSe analysis showed that a total of 13 bacteria had significant changes across the control group, DSS group, and EBDEV-treated group. (Figure 7R). The evolutionary branching diagram shows the major microorganisms in each group, with the diameter size of the circles on each stratum proportional to the relative abundance. Characteristic bacteria of the control group included *Rikenellaceae*, *Roseburia*, *Muribaculaceae*, *Alistipes*, and *Eubacterium_xylanophilum*. Characteristic bacteria of the DSS group included *Romboutsia*, *Erysipelotrichales*, and *Peptostreptococcales*. Characteristic bacteria of the EBDEV group included *Proteobacteria*, *Eubacterium_fissicatena*, and *Gammaproteobacteria* (Figure 7Q).

## 4. Discussion

**Figure 8:**
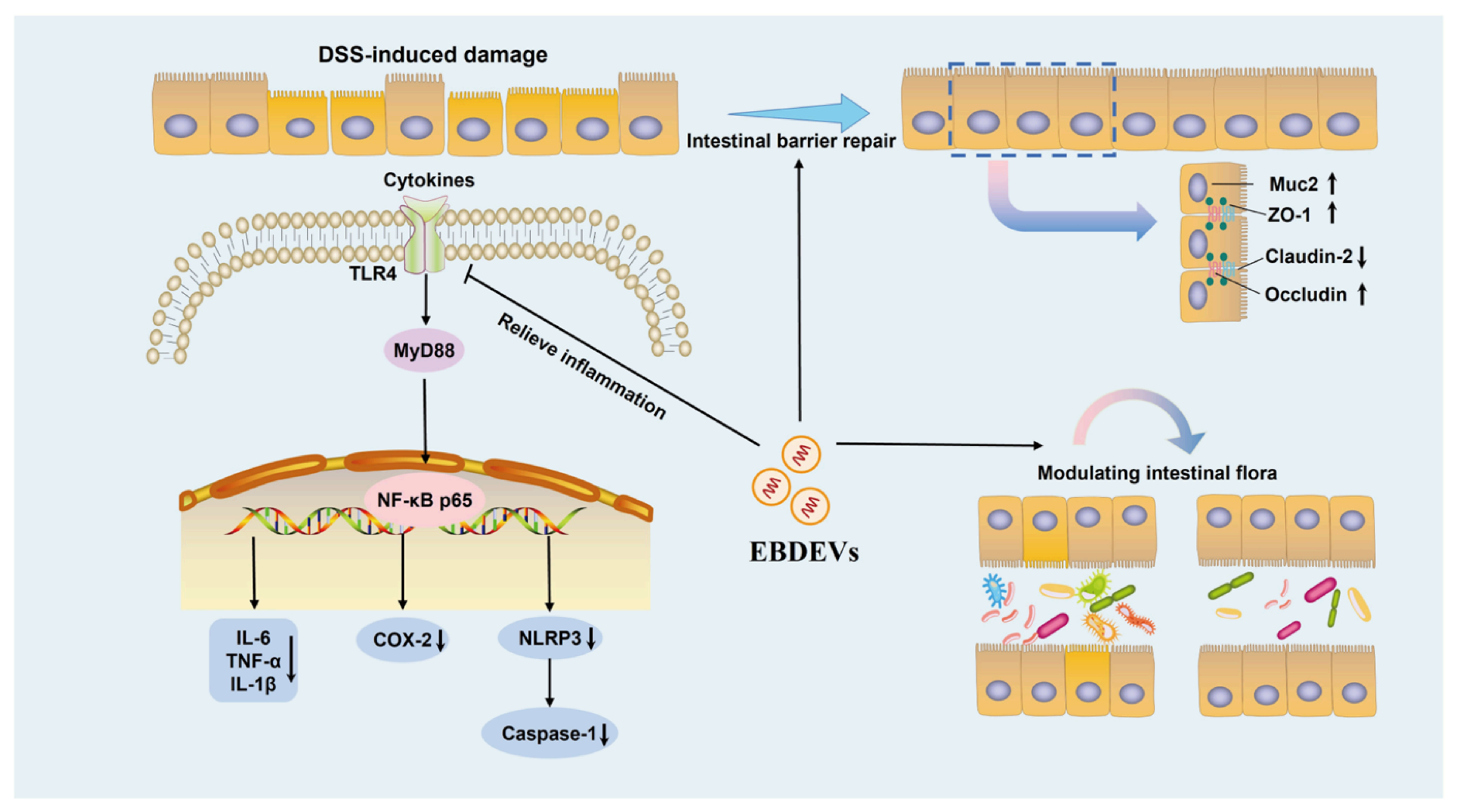
Mechanism diagram of EBDEV in alleviating UC.

UC is a refractory chronic intestinal inflammatory disease, which currently has therapeutic options with considerable limitations in terms of efficacy, safety, and cost [27]. A number of PDEVs are natural nanoscale vesicles with high safety, good biocompatibility, and low immunogenicity, and exert integrated therapeutic effects, including anti-inflammation, barrier repair, and microbiota regulation, providing a novel strategy for UC treatment. Preclinical studies have verified the anti-colitis effects of PDEVs from *ginseng*, *Centella asiatica*, and *Houttuynia cordata*, regulating immune imbalance, repairing the epithelial barrier, and improving gut microbiota homeostasis [3,28,29]. In this study, we successfully isolated and characterized extracellular vesicles from *Elaeocarpus braceanus* fruits and systematically evaluated their therapeutic effects and underlying mechanisms of inducing remission of UC (Figure 8).

Isolation and purification methods are fundamental for obtaining high-quality PDEVs [30]. This study employed differential centrifugation combined with sucrose density gradient ultracentrifugation to obtain EBDEVs, a classic method for isolating plant-derived extracellular vesicles offering high purification efficiency [31]. Our results indicated that EBDEVs exhibited a typical spherical morphology, with an average hydrodynamic particle size of approximately 187 nm, a PDI of 0.104, a negative surface charge, a high particle concentration of 8.1 × 10^11^ particles/mL, and high stability under storage conditions, consistent with characteristics reported in existing PDEVs research [32,33]. The increased particle size of EBDEVs in SIF is presumed to stem from the destruction of vesicle membrane structures induced by SIF. Impaired membrane integrity facilitates the fusion of adjacent vesicles, which eventually aggregates into larger particles [34]. The naturally active substances identified in metabolomics analysis further constitute the material basis for the biological activity of EBDEVs. For instance, flavonoids like kaempferol and quercetin have antioxidant and anti-inflammatory effects by scavenging reactive oxygen species and inhibiting the release of inflammatory factors [35,36]; tannins such as corilagin and gallic acid also contribute to antioxidant effects and protect the intestinal mucosal barrier [37,38].

In vitro experiments confirmed that EBDEVs could be effectively taken up by RAW264.7 macrophages, indicating their potential to regulate intestinal immune cell function. At non-cytotoxic concentrations, EBDEVs significantly suppressed LPS-induced NO production in macrophages, demonstrating remarkable in vitro anti-inflammatory activity and the ability to modulate macrophage-mediated inflammatory cascades.

Currently, the main quantitative method used for PDEVs is through total protein quantification. The dosage used for animal experiments ranges from 0.1 to 100 mg total protein/kg body weight [39]. Our experiment selected 5 and 15 mg total protein/kg body weight as the gavage dosages for animals. Mice in the model group displayed typical UC-related symptoms, including weight loss, elevated DAI scores, shortened colon, and colonic congestion, edema, and erosion, confirming successful model establishment. EBDEVs treatment could alleviate these symptoms. Serum inflammatory cytokine detection represents a critical indicator for evaluating systemic inflammation in UC [40]. EBDEVs reduced serum levels of IL-6, TNF-α, IFN-γ, and IL-1β in UC mice. As core pro-inflammatory cytokines in UC pathogenesis, these factors drive mucosal inflammation, immune cell infiltration, and tissue damage [41]. H&E staining confirmed that EBDEVs can ameliorate the pathological damage in UC mice.

Intestinal barrier repair is a critical strategy for the therapy of UC [42]. The intestinal barrier is composed of the mucus barrier and the epithelial tight junction barrier. Impairment of either barrier can elevate intestinal permeability and cause bacterial translocation, which in turn activates inflammatory responses [43]. Goblet cells play a key role in the synthesis and secretion of mucins [44]. Muc2 is the most abundantly secreted mucin in the gastrointestinal tract and constitutes the core component of the mucus barrier [45]. Our results indicate that the administration of EBDEVs can ameliorate the damage to goblet cells and suggest that EBDEV may be able to increase the content of mucin Muc2 and restore the mucus barrier in UC mice. The effect of longer treatment with EBDEVs needs to be explored in future studies. Tight junction proteins form the core of the epithelial barrier. ZO-1 protein influences barrier repair efficacy [46]. Occludin closes the paracellular pathway, constituting the fundamental structure of tight junctions [47]. Claudin-2 is a pore-forming protein that disrupts the tight junction barrier and is dysregulated in many pathological processes, including inflammation, cancer, and fibrosis [48]. Our results indicate that EBDEVs can significantly upregulate the expression of ZO-1 and downregulate the expression of Claudin-2. While the expressions of Muc2 and Occludin after EBDEV treatments showed no significant difference with the DSS group, the trends of their average expression levels suggested that continued treatment with EBDEV may restore expression of these proteins to control levels. As noted above, the effect of longer treatment with EBDEVs needs to be explored in future studies.

The TLR4/MyD88/NF-κB signaling pathway is a classic pro-inflammatory pathway in UC pathogenesis, whose aberrant activation is central to the perpetuation of the UC inflammatory response [49]. DSS can activate the TLR4/MyD88/NF-κB pathway by damaging the intestinal barrier and activating immune cells, leading to NF-κB p65 nuclear translocation, which in turn initiates the transcription of downstream pro-inflammatory cytokines (e.g., IL-6, IL-1β) and inflammatory mediators (e.g., COX-2) [50]. Furthermore, activation of this pathway can further induce NLRP3 inflammasome activation (via Caspase-1 activation), amplifying the inflammatory cascade [51]. Our results confirm that the anti-inflammatory mechanism of EBDEVs is to block the initiation and amplification of inflammatory responses by inhibiting the activation of the TLR4/MyD88/NF-κB signaling pathway and the consequent activation of the NLRP3 inflammasome (Figure 8).

Gut microbiota dysbiosis is a critical inducer and regulatory factor in the pathogenesis of UC, and restoring gut microbiota homeostasis constitutes an important therapeutic strategy for UC [52]. Previous studies have demonstrated that DSS triggers marked gut microbiota dysbiosis, characterized by disrupted microbial diversity, reduced beneficial bacteria, and increased pro-inflammatory bacteria [53]. In this study, EBDEV intervention restored gut microbiota α-diversity, indicating its ability to alleviate DSS-induced gut microbiota dysbiosis. Partial least squares discriminant analysis (PLS-DA) based on operational taxonomic units (OTUs) and beta diversity principal coordinate analysis based on weighted UniFrac distances further confirmed that EBDEVs exert regulatory effects on the gut microbiota composition in UC mice. At the phylum level, gut microbiota composition analysis revealed that EBDEVs corrected the imbalance of dominant bacterial phyla caused by DSS. At the genus level, the beneficial genus *Lachnospira* produces short-chain fatty acids (SCFAs) and helps maintain intestinal barrier function [54]. In contrast, *Prevotella* and *Bacteroides* proliferate abnormally in UC and elicit intestinal inflammation [55]; moreover, *Akkermansia* is closely linked to mucus barrier damage [56]. EBDEVs administration normalized the levels of all the above-mentioned bacteria in this study. LEfSe analysis further identified characteristic bacteria for each group: the control group was enriched in beneficial bacteria such as *Rikenellaceae* [57] and *Roseburia* [58]; the DSS group was characterized by harmful bacteria like *Erysipelotrichales* [59] and *Romboutsia* [60]; and the EBDEV-treated group was characterized by bacteria such as *Gammaproteobacteria* [61], which are associated with the regulation of intestinal inflammation and barrier function. These results confirm that EBDEVs can reshape the intestinal flora composition of UC mice and promote intestinal barrier repair to alleviate intestinal inflammatory stimulation and form a positive feedback loop with the anti-inflammatory effects of EBDEVs.

Multiple PDEVs have demonstrated therapeutic potential against IBD. Turmeric-derived exosome-like nanovesicles (TDNPs) suppress pro-inflammatory cytokines (TNF-α, IL-6, IL-1β) and upregulate HO-1 via NF-κB pathway inactivation and are preferentially internalized by colonic epithelial cells and macrophages [62]. Ginseng-derived nanoparticles (GDNPs) exert dual anti-inflammatory and antioxidant effects by inhibiting the TLR4/MAPK pathway while activating the p62/Nrf2/Keap1 axis and additionally promote intestinal stem cell proliferation via Wnt/β-catenin signaling [28]. Broccoli-derived nanoparticles (BDNs) induce tolerogenic dendritic cells through AMPK activation, with sulforaphane (SFN) identified as a key bioactive component responsible for this immunomodulatory effect [63]. Compared with these PDEVs, EBDEVs exhibit several distinctive features. They are rich in flavonoids, terpenoids, and tannins, which collectively contribute to their multi-targeted pharmacological actions. Notably, EBDEVs not only restore the intestinal barrier but also inhibit the TLR4/MyD88/NF-κB signaling pathway and downstream NLRP3 inflammasome activation, while modulating gut microbiota composition. This synergistic multi-pathway mechanism endows EBDEVs with unique therapeutic advantages. Furthermore, to the best of our knowledge, this is the first study to demonstrate the anti-ulcerative colitis activity of EBDEVs, thereby expanding the repertoire of plant-derived extracellular vesicles with therapeutic potential for UC.

## 5. Conclusions

In this study, extracellular vesicles derived from *Elaeocarpus braceanus* fruits were successfully isolated and characterized. In vitro experiments demonstrated that EBDEVs could be taken up by macrophages and significantly inhibited LPS-induced NO production, confirming their anti-inflammatory activity in vitro. In vivo, EBDEV administration ameliorated body weight loss and diarrhea, and alleviated colonic tissue damage in UC mice. The mechanistic studies revealed that the protective effects of EBDEVs against UC are primarily attributed to the restoration of the intestinal barrier, inhibition of the TLR4/MyD88/NF-κB signaling pathway, and modulation of the gut microbiota. These findings provide experimental basis and theoretical support for the development of plant-derived extracellular vesicles as nature nanoparticles and delivery systems for inflammatory bowel disease therapy.

## Data Availability

The datasets used and/or analyzed during the current study are available from the corresponding author upon reasonable request.

## Supplementary Materials

The following supporting information can be downloaded at: https://www.mdpi.com/article/10.3390/nano16150968/s1. Supplementary Materials S1: Table S1: Formula for Simulated Gastric Fluid and Simulated Intestinal Fluid; Table S2: Criteria for scoring DAI; Table S3: The scoring standard of histopathological analysis; Table S4: Primer sequences for quantitative real-time PCR reactions; Table S5: Some natural products in EBDEVs were identified by untargeted metabolomics and Complete unedited protein Western blotting images. Supplementary Materials S2: Components in EBDEVs were identified by untargeted metabolomics.

## Abbreviations

EBDEVs, Extracellular vesicles derived from *Elaeocarpus braceanus*; PDEVs, Plant-derived extracellular vesicles; UC, Ulcerative colitis; LPS, lipopolysaccharide; DSS, Dextran Sulfate Sodium Salt; 5-ASA, 5-aminosalicylic acid; TEM, Transmission Electron Microscope; NTA, Nanoparticle Tracking Analysis DAI, disease activity index; H&E, hematoxylin and eosin; IL-6, interleukin-6; TNF-α, tumor necrosis factor-α; IFN-γ, interferon-γ; IL-1β, interleukin-1β; AB-PAS, Alcian blue-periodic acid-Schiff; TLR4, Toll-like receptor 4; MyD88, Myeloid Differentiation Primary Response 88; NF-κB, nuclear factor kappa-B; COX-2, Cyclooxygenase-2; NLRP3, NOD-like receptor thermal protein domain associated protein 3; Caspase-1, cysteinyl aspartate specific proteinase-1; PCA, Principal Component Analysis; LEfSe, Linear discriminant analysis effect size.
