# Supplementary material for: Extracellular Vesicles Derived from Elaeocarpus braceanus Alleviate DSS-Induced Ulcerative Colitis in Mice Through Multiple Pathways
Source: Nanomaterials (Basel). 2026 Aug 6;16(15):968. doi: 10.3390/nano16150968 (PMC13468697; doi:10.3390/nano16150968)

Extracellular vesicles derived from *Elaeocarpus braceanus* alleviate DSS-induced ulcerative colitis in mice through multiple pathways

Wen-Bo Feng[1], Tong Liu[1], Mu-Yao Liu[1], Hui-Ying Fu[2], Lu Li[1], Zheng-Yi Zhou[1], Qiang Cai[2*], Yu-Xin Chen[1*]

[1] Hubei Key Laboratory of Industry Microbiology, Key Laboratory of Fermentation Engineering (Ministry of Education), School of Life and Health Sciences, Hubei University of Technology, Wuhan 430068, China

[2] State Key Laboratory of Hybrid Rice, Hubei Hongshan Laboratory, College of Life Sciences, Wuhan University, Wuhan 430072, China

Supplementary Tables

Table S1. Formula for Simulated Gastric Fluid and Simulated Intestinal Fluid.

Name	Formula
Simulated Gastric Fluid(SGF), pH 1.2	Hydrochloric acid, 0.01 g/mL Pepsin
Simulated Intestinal Fluid(SIF), pH 6.8	20% 0.1 mol/L Potassium Dihydrogen Phosphate, 80% 0.1 mol/L Dipotassium Hydrogen Phosphate, 2% Trypsin

Table S2. Criteria for scoring DAI.

**Score**	**Rate of Weight Change**	**Stool Formation**	**Occult Blood Status**
0	0%	Normal	No color development within 2 min
1	1%-5%	Soften	Change from light green to green after 10 s
2	5%-10%	Loose	Change from light green to blue－brown
3	10%-15%	Unshaped	Change from blue－brown to black－brown
4	>15%	Diarrhea	Immediately turns blue－black－brown

* Corresponding author: yuxinc@hbut.edu.cn; qiang.cai@whu.edu.cn

Table S3. The scoring standard of histopathological analysis.

Scores	The morphology of colon tissue
0	Normal
1	Mild damage to the crypt, slight inflammatory cell infiltration
2	Moderate damage to the crypt, partial inflammatory cell infiltration
3	Severe damage to the crypt, massive inflammatory cells infiltrating, intact epithelium
4	Severe damage to the crypt, massive inflammatory cells infiltrating, epithelial loss

Table S4. Primer sequences for quantitative real-time PCR reactions.

Gene name	Primer sequence
β-actin	Forward 5′-GCAGGAGTACGATGAGTCCG-3′<br>Reverse 5′-ACGCAGCTCAGTAACAGTCC-3′
Muc2	Forward 5′-CCTGAAGACTGTCGTGCTGT-3′<br>Reverse 5′-CAGCGTAGTTGGCACTCTCA-3′
ZO-1	Forward 5′-GCCGCTAAGAGCACAGCAA-3′<br>Reverse 5′-GCCCTCCTTTTAACACATCAGA-3′
Occludin	Forward 5′-GTGAATGGGTCACCGAGGG-3′<br>Reverse 5′-AGATAAGCGAACCTGCCGAG-3′
Claudin-2	Forward 5′-TGCGACACACAGCACAGGCATCA-3′<br>Reverse 5′-TCAGGAACCAGCGGCGAGTAGAA-3′
TLR4	Forward 5′-ATGGCATGGCTTACACCACC-3′<br>Reverse 5′-GAGGCCAATTTTGTCTCCACA-3′
MyD88	Forward 5′-TCATGTTCTCCATACCCTTGGT-3′<br>Reverse 5′-AAACTGCGAGTGGGGTCAG-3′
NF-κB p65	Forward 5′-ATGATCGCCACCGGATTGAA-3′<br>Reverse 5′-AAGGACTTCCTTACCTGGCTTG-3′
COX-2	Forward 5′-TGCTGTACAAGCAGTGGCAA-3′<br>Reverse 5′GCAGCCATTTCCTTCTCTCC-3′
NLRP3	Forward 5′-TGGGTTCTGGTCAGACACGAG-3′<br>Reverse 5′-ATCCAGACGTATGTCCTGAGC-3′
Caspase-1	Forward 5′-TCTGTATTCACGCCCTGTTGGAAAG-3′<br>Reverse 5′CTCTTTGCCCTCAGGATCTTGTCAG-3′

Table S5. Some natural products in EBDEVs were identified by untargeted metabolomics.

Serial Number	Alignment ID	Metabolite name	Rt(min)	Expreiment Mz	Class
1	POS9462	Kaempferol	6.099	287.05347	Flavonoid
2	NEG16953	Myricetin 3-Xyloside	4.487	449.07187	Flavonoid
3	NEG15690、POS14985	Shoyuflavone C	4.915	417.04514	Flavonoid
4	POS16074	3'-O-Methylderhamnosylmaysin	6.34	445.11215	Flavonoid
5	NEG16955	Aromadendrin 4'-glucoside	4.964	449.10797	Flavonoid
6	NEG22344	Quercetin 3-O-sambubioside	6.476	594.99933	Flavonoid
7	NEG17637	6-C-Glucopyranosyldihydroquercetin	4.808	465.10281	Flavonoid
8	NEG10981	Glyzarin	6.979	293.08121	Flavonoid
9	POS16228	Dichotosin	6.451	449.17645	Flavonoid
10	POS16884、NEG17551	Myricitrin	5.564	465.10159	Flavonoid
11	POS17544	Herbacetin 8-glucuronide	5.475	479.0813	Flavonoid
12	NEG16859	Quercitrin	5.73	447.09271	Flavonoid
13	NEG18163	Myricetin 3-galactoside	5.171	479.08096	Flavonoid
14	NEG18089	Quercetin-4'-glucuronide	5.322	477.0657	Flavonoid
15	POS15563、NEG16211	Kaempferol 3-Rhamnoside	6.099	433.11206	Flavonoid
16	POS11500	7,2',3'-Trimethoxyflavanone	7.332	337.10425	Flavonoid
17	NEG11302	Quercetin	4.182	301.03772	Flavonoid
18	POS17801	Termopsoside	7.924	485.1123	Flavonoid

Continued Table

Serial Number	Alignment ID	Metabolite name	Rt(min)	Expreiment Mz	Class
19	NEG15289	6-Geranylnaringenin	8.667	407.18546	Flavonoid
20	POS10806	Myricetin	5.559	319.04453	Flavonoid
21	NEG11932	Dihydromyricetin	5.246	319.04495	Flavonoid
22	NEG19791	Mascaroside	5.402	523.21588	Flavonoid
23	POS8251	Piscidein	4.833	257.06503	Flavonoid
24	NEG14414	Icariside H1	5.765	385.14899	Flavonoid
25	NEG13410	Semperoside	5.322	359.13422	Flavonoid
26	NEG11296、POS10118	Ellagic acid	5.487	300.99799	Tannins
27	POS10878、NEG11929	Luteic acid	4.979	321.02341	Tannins
28	POS10705、NEG11795	3-O-Methylellagic acid	5.593	317.02872	Tannins
29	NEG17786	Valoneic acid dilactone	4.382	469.00394	Tannins
30	POS11856、NEG12801	3,4,3'-Tri-O-methylellagic acid	6.175	345.05978	Tannins
31	POS17175	Sanguisorbic acid dilactone	5.247	471.01718	Tannins
32	NEG13407	Syringic acid beta-glucopyranosyl ester	4.262	359.09705	Tannins
33	NEG16290、POS15634	Ellagic acid-4-O-beta-D-xylopyranoside	5.15	433.04013	Tannins

Continued Table

Serial Number	Alignment ID	Metabolite name	Rt(min)	Expreiment Mz	Class
34	POS16801、NEG17464、POS17800	6-Cinnamoyl-1-galloylglucose	6.265	463.12219	Tannins
35	NEG23050	2-Cinnamoyl-1,6-digalloyl-beta-D-glucopyranose	6.287	613.11859	Tannins
36	NEG23049	6-Cinnamoyl-1,2-digalloylglucose	6.492	613.11816	Tannins
37	NEG23861	1,4,6-Trigalloyl-beta-D-glucopyranose	4.93	635.0885	Tannins
38	NEG28810	1,2,3,4-Tetragalloyl-alpha-D-glucose	5.26	787.09937	Tannins
39	POS17601	NCGC00380690-01_C22H22O11_beta-D-Glucopyranose, 6-O-[(2E)-1-oxo-3-phenyl-2-propen-1-yl]-, 1-(3,4,5-trihydroxybenzoate)	6.265	480.15012	Tannins
40	NEG17032	3,4-Hexahydroxydiphenoylarabinose	4.21	451.05054	Tannins
41	NEG23778	Phyllanemblinin B	4.725	633.07043	Tannins
42	POS23826、NEG23698	Castalin	5.221	633.07068	Tannins
43	POS24029	Gallotannin	5.147	637.10046	Tannins

Continued Table

Serial Number	Alignment ID	Metabolite name	Rt(min)	Expreiment Mz	Class
44	NEG18317、POS18487、POS17798	1,6-di-O-Galloylglucose	4.42	483.07651	Tannins
45	NEG23779	Isocorilagin	4.224	633.07068	Tannins
46	NEG23781	Corilagin	5.215	633.07477	Tannins
47	POS23921	Strictinin	5.186	635.08466	Tannins
48	POS23199、NEG23116	Phyllanemblinin A	5.263	617.07599	Tannins
49	POS31694	Nobotanin D	5.618	787.09406	Tannins
50	NEG29133	lagerstannin A	4.377	799.06183	Tannins
52	POS5482、NEG6910	Methyl gallate	5.191	185.04414	Tannins
53	NEG12364	2-Galloylglucose	3.652	331.06647	Tannins
54	POS18698	Hamamelitannin	4.887	507.07315	Tannins
55	NEG18645	1'-O-Galloylsucrose	4.13	493.11893	Tannins
56	NEG12580	Dehydrodigallensaeure	6.221	337.01877	Tannins
57	NEG24457	chebulanin	4.452	651.08386	Tannins
58	POS32328	granatin A	5.263	801.07373	Tannins
59	POS7016	Adalinine	6.68	226.17932	Alkaloids
60	POS14792	Echimidine N-oxide	7.146	414.21362	Alkaloids
61	POS9474	13-Hydroxylupanine	6.305	287.17462	Alkaloids
62	POS11263	1-O-Acetyllycorine	4.967	330.13208	Alkaloids
63	POS3814	Arecaidine	4.809	142.08598	Alkaloids

Continued Table

Serial Number	Alignment ID	Metabolite name	Rt(min)	Expreiment Mz	Class
64	POS9027	1-(3,4-Dimethoxycinnamoyl)piperidine	7.426	276.15854	Alkaloids
65	POS12529	Scopolamine-N-butyl	7.21	360.21329	Alkaloids
66	POS12768	7-(2'-Hydroxy-3'-chloroprenyloxy)-4,8-dimethoxyfuroquinoline	4.887	366.11038	Alkaloids
67	POS3649	Trigonelline	0.912	138.05449	Alkaloids
68	POS6665	Tschimganidin	4.554	217.10449	Alkaloids
69	POS5724	Diethyltoluamide	6.944	192.13757	Alkaloids
70	POS3737、POS2682	Betaine	0.875	140.06801	Alkaloids
71	POS6662	Lycoperodine 1	4.713	217.09682	Alkaloids
72	NEG17948	Peperomin F	6.329	473.1445	Alkaloids
73	POS8588	Subaphylline	4.761	265.1539	Alkaloids
74	POS6918	Gardenine	6.808	224.0912	Alkaloids
75	POS8764	3,5-Hexalobine B	7.554	270.18448	Alkaloids
76	POS11097	Broussonetine W	7.351	326.23166	Alkaloids
77	POS11557	Loesenerine	9.014	338.28302	Alkaloids
78	NEG12225	Paeonoside	4.896	327.10727	Terpenoids
79	POS3617	gamma-Terpinene	4.364	137.13223	Terpenoids
80	POS15415	Cichorioside N	5.524	429.21054	Terpenoids
81	POS17483	Hongdoushan B	6.295	477.28391	Terpenoids
82	POS15484	Hierapolitanin D	6.325	431.22672	Terpenoids

Continued Table

Serial Number	Alignment ID	Metabolite name	Rt(min)	Expreiment Mz	Class
83	POS5758	(-)-gamma-ionone	5.892	193.15826	Terpenoids
84	POS6283	3-Keto-beta-ionone	6.974	207.13766	Terpenoids
85	POS16729	Hyperielliptone HA	6.605	461.28879	Terpenoids
86	POS9393	Hysterone D	5.52	285.16824	Terpenoids
87	NEG12219	(E)-2-Methylgeranyl diphosphate	4.901	327.07077	Terpenoids
88	NEG18784	Globulusin B	5.76	497.16553	Terpenoids
89	NEG14827	Australin A	7.039	395.24323	Terpenoids
90	POS16077	Valtrate	5.964	445.18103	Terpenoids
91	POS17720	Kadsulactone A	6.491	483.30838	Terpenoids
92	POS16158	Dendroside G	5.52	447.22159	Terpenoids
93	NEG12805	Teuhircoside	4.257	343.10254	Terpenoids
94	NEG18095	Eremopetasitenin D2	5.502	477.19699	Terpenoids
95	POS12734	4alpha-Phorbol	7.637	365.19226	Terpenoids
96	POS16548	Dendronpholide L	7.533	457.27499	Terpenoids
97	POS25484	Steviobioside	6.641	665.31183	Terpenoids
98	POS16474	Incaspitolide E	6.035	455.2229	Terpenoids
99	POS5761	gamma-Ionone	5.728	193.16087	Terpenoids
100	POS5486	Chokol G	7.215	185.15343	Terpenoids
101	NEG24882	Azadirachtin P	5.779	663.26654	Terpenoids
102	POS18379	Euphohelioscopin A	6.714	499.3046	Terpenoids
103	POS9312	Qing Hau Sau	6.37	283.15103	Terpenoids
104	POS9069	Buddledin A	7.575	277.17877	Terpenoids

Continued Table

Serial Number	Alignment ID	Metabolite name	Rt(min)	Expreiment Mz	Class
105	POS3619	(S)-alpha-Phellandrene	1.285	137.13223	Terpenoids
106	POS23682	Resiniferatoxin	9.337	629.26984	Terpenoids
107	POS22164	Trigonosin A	8.561	591.25757	Terpenoids
108	POS20285	Brusatol	5.999	543.18237	Terpenoids
109	POS16436	Fuziline	6.431	454.28262	Terpenoids
110	NEG17556	Rosiridoside B	5.366	463.2179	Terpenoids
111	POS5760	beta-Ionone	5.633	193.15829	Terpenoids
112	NEG14482	Ixoside	4.266	387.09204	Terpenoids
113	POS17721	Coronalolide	6.219	483.30872	Terpenoids
114	NEG20913	Abelioside A	4.935	553.19171	Terpenoids
115	POS17646	Soyasapogenol B	7.973	481.36447	Terpenoids
116	POS9647	Madolin D	6.205	291.15823	Terpenoids
117	NEG15122	7,8-Didehydropenstemoside	4.516	403.12274	Terpenoids
118	NEG19267	Linalool 3,7-oxide beta-primeveroside	5.126	509.22134	Terpenoids
120	POS15697	Anthriscifolcine B	6.641	436.27087	Terpenoids
121	POS14408	10-Acetylmonomelittoside	4.818	405.13727	Terpenoids
122	POS11298	Carnosol	7.347	331.18713	Terpenoids
123	POS12908	Cedronolactone A	5.196	369.18924	Terpenoids
124	POS3463、POS3462	p-Mentha-1,3,5,8-tetraene	5.848	133.10092	Terpenoids
125	NEG9433	Aplysinopsin	6.881	253.10715	Terpenoids
126	POS10888	Pseudoanisatin	5.892	321.13007	Terpenoids

Continued Table

Serial Number	Alignment ID	Metabolite name	Rt(min)	Expreiment Mz	Class
127	POS14568	Eudesmin	7.018	409.16141	Terpenoids
128	POS8733	Arteincultone	6.325	269.17416	Terpenoids
129	POS12300	Euphorbetin	5.634	355.04343	Terpenoids
130	NEG14488	Swertiajaposide A	4.648	387.13535	Terpenoids
131	POS2976	Santene	5.242	123.11686	Terpenoids
132	POS9265	Rhodiocyanoside A	5.368	282.09677	Terpenoids
133	POS9644	Aurin	2.02	291.10413	Organic acid
134	POS17381	m-Trigallic acid	4.911	475.04941	Organic acid
135	NEG7869、POS6422	Jasmonic acid	6.293	209.11707	Organic acid
136	NEG11866	Flavellagic acid	5.443	316.99268	Organic acid
137	POS11783	Lauramidopropylbetaine	6.758	343.29425	Organic acid
138	POS4950、POS4195、POS4198、NEG6355	Gallic acid	3.581	171.02829	Organic acid
139	NEG9865	Abscisic acid	6.451	263.12808	Organic acid
140	NEG6097	4-Hydroxycinnamic acid	5.438	163.03857	Organic acid
141	POS3375、POS4083	Cinnamic acid	5.818	131.04892	Organic acid
142	POS7831	Abscisic acid	5.694	247.13179	Organic acid
143	POS8580	Purpurogallincarboxylic acid	5.938	265.0332	Organic acid
144	NEG13250	(+)-Chebulic acid	4.168	355.02872	Organic acid

Continued Table

Serial Number	Alignment ID	Metabolite name	Rt(min)	Expreiment Mz	Class
145	NEG8429	Glucoheptonic acid	0.89	225.06047	Organic acid
146	NEG12795	Fujikonaic acid	5.683	343.00916	Organic acid
147	POS5751、POS6578	Citric acid	2.185	193.03371	Organic acid
148	POS10367	Corchorifatty acid D	7.27	309.20523	Organic acid
149	POS9878	Phaseolic acid	4.887	297.05978	Organic acid
150	POS6242	Xanthurenic acid	4.612	206.04437	Organic acid
151	POS9553	Tragopogonic acid	5.171	289.06937	Organic acid
152	NEG3470	Maleic acid	0.959	115.00192	Organic acid
153	NEG7708	Homocitric acid	4.182	205.03363	Organic acid
154	POS11272	Morusimic acid F	6.579	330.26288	Organic acid
155	POS11292、POS11293	Miraxanthin III	4.833	331.12714	Organic acid
156	NEG4662	Malic acid	1.347	133.01242	Organic acid
157	POS6968	Apiosporic acid	6.974	225.14783	Organic acid
158	POS3307	Pipecolic acid	1.566	130.08607	Organic acid
159	POS3694	Fumaric acid	2.175	139.00238	Organic acid
160	NEG5389	Citramalic acid	2.203	147.02798	Organic acid
161	POS5400	Homovanillic acid	4.584	183.065	Organic acid
162	POS5161	Ferulic acid	5.114	177.05415	Organic acid
163	NEG32453	Chebulagic acid	4.275	953.08582	Organic acid
164	NEG11220	Emodic acid	5.683	299.01816	Organic acid

Continued Table

Serial Number	Alignment ID	Metabolite name	Rt(min)	Expreiment Mz	Class
165	POS11868、POS11136、NEG13498	Secoisolariciresinol	5.553	345.16916	Lignans
166	POS12481	Matairesinol	5.999	359.14728	Lignans
167	NEG13498	Secoisolariciresinol	8.166	361.16495	Lignans
168	NEG14972	Kynapcin-28	6.091	399.03366	Lignans
169	NEG9924	Kynapcin-13	5.15	265.03412	Lignans
170	NEG15055	Ulexin B	5.438	401.14362	Coumarins
171	NEG6995	Angelicin	0.89	185.02068	Coumarins
172	POS11295	Fragransol B	5.49	331.15265	Others
173	NEG10422	Neodunol	7.224	279.0658	Others
174	NEG15434	Turgorin	4.163	411.02289	Others
175	POS11294	Biflorin (Capraria)	5.332	331.12833	Others
176	NEG10345、POS9136	Nepenthone A	6.588	277.03418	Others
177	NEG19263	Mallophenol A	4.72	509.12845	Others
178	NEG12218	bergenin	4.569	327.07074	Others
179	NEG10041	Emodin	7.29	269.04437	Others
180	POS6920	Maokonine	4.664	224.12762	Others
181	POS10630、NEG11729	Norbergenin	4.892	315.07022	Others
182	NEG15608	Phenylethyl primeveroside	6.086	415.15952	Others

Continued Table

Serial Number	Alignment ID	Metabolite name	Rt(min)	Expreiment Mz	Class
183	POS8799	(1R*,2R*,3R*,4aS*,5R*)-octahydro-3-(3-hydroxyprop-1-en-2-yl)-4a,5-dimethylnaphthalene-1,2,8a(1H)-triol|tuberculariol C	7.023	271.18741	Others
184	NEG9858	Ancistroquinone E	5.804	263.01895	Others
185	NEG12365	1-O-Galloylfructose	3.394	331.0665	Others
186	POS17809	Ardisiaquinone E	7.909	485.2872	Others
187	POS6221	Cladoacetal A	7.669	205.08562	Others
188	NEG25678	Kidamycin	6.508	687.31976	Others
189	POS10185	12-Methoxycitromycetin	4.863	305.06494	Others
190	POS2741	Indane	7.16	119.08545	Others
191	NEG7195	Ibufenac	2.219	191.10359	Others
192	POS7052	Ficusol	4.937	227.09082	Others
193	POS9712	Phyllanthusiin E	4.987	293.02826	Others
194	POS7907	Brevifolin[Geranium]	5.52	249.04025	Others
195	NEG10895	Phyllanthusiin E	5.669	291.01367	Others
196	POS18540	Platanionoside F	5.524	503.24768	Others
197	NEG18446	Cistanoside F	4.248	487.14481	Others
198	NEG15788	Euodionoside E	5.863	419.22668	Others
199	NEG10123	Moracin J	7.279	271.06042	Others
200	NEG19185	Clemastanin A	4.939	507.1868	Others
201	NEG19181	Moghanin A	5.589	507.11362	Others

Continued Table

Serial Number	Alignment ID	Metabolite name	Rt(min)	Expreiment Mz	Class
202	POS8767	Capsiamide	8.679	270.27817	Others
203	POS2491	Tulipalin B	9.778	115.03896	Others
204	POS8170	Russulanorol	6.054	255.15834	Others
205	NEG9927	Typharin	6.919	265.08615	Others
206	POS10489	Tetrahydropiperine	5.923	312.16141	Others
207	POS16840	Rosellichalasin	6.205	464.23911	Others
208	NEG5392	p-Coumaraldehyde	6.086	147.04353	Others
209	NEG32409	Acalyphidin M2	5.081	951.07166	Others
210	NEG16291	ducheside B	4.347	433.04013	Others
211	POS9062	Dihydromethysticin	4.972	277.10632	Others
212	NEG15054	lamiophlomiside	5.654	401.14359	Others
213	POS13909	Fraxin	0.757	393.08136	Others
214	POS14213	Kynapcin-28	4.717	401.04825	Others
215	POS16973	4-O-galloylbergenin|4-O-Galloylnorbergenin	5.137	467.08145	Others
216	NEG32410	carpinusin	4.787	951.07306	Others
217	NEG18238	Myrciaphenone B	4.42	481.09631	Others
218	POS18468	Tomoeone B	6.219	501.32108	Others
219	NEG15348	3,3'-biflaviolin	6.621	409.01865	Others
220	POS4776	Dihydrojasmone	7.363	167.14273	Others
221	POS16975	11-O-galloylbergenin|11-O-Galloylnorbergenin|6-O-galloylnorbergenin	4.636	467.08176	Others

Continued Table

Serial Number	Alignment ID	Metabolite name	Rt(min)	Expreiment Mz	Class
222	POS12205	Epijasminoside A	6.34	353.15549	Others
223	POS6872	Fraxidin	1.496	223.06229	Others
224	POS9148	Cernuine N-oxide	6.574	279.20294	Others
225	NEG15992	Naucleidinal	6.381	425.18134	Others
226	POS12948	Aplidiasphingosine	8.144	370.3306	Others
227	NEG8891	Micrandrol A	7.061	237.09131	Others
228	NEG11676	Triptophenolide	8.113	311.16803	Others
229	NEG7410	Ethyl gallate	5.563	197.04401	Others
230	POS6428	alpha-Cyclogeraniol acetate	6.93	211.16881	Others
231	POS7909	Dendrodolide N	6.609	249.10927	Others
232	NEG9093	Pandangolide 1a	5.877	243.12289	Others
233	NEG8519	Cladospolide B	6.293	227.12805	Others
234	NEG11997	Digallate	5.27	321.0242	Others
235	POS6309	Murrayaculatine	4.765	208.05977	Others
236	NEG17868	(2R,3R)-dihydromyricetin-4'-O-gallate	5.774	471.05524	Others
237	POS6048	Stagonolide G	4.347	201.11154	Others
238	NEG8049	Bergapten	0.871	215.03174	Others

Note: Alignment ID is the molecular ion peak ID

Complete unedited protein Western blotting images

1. TLR4 (95kDa)

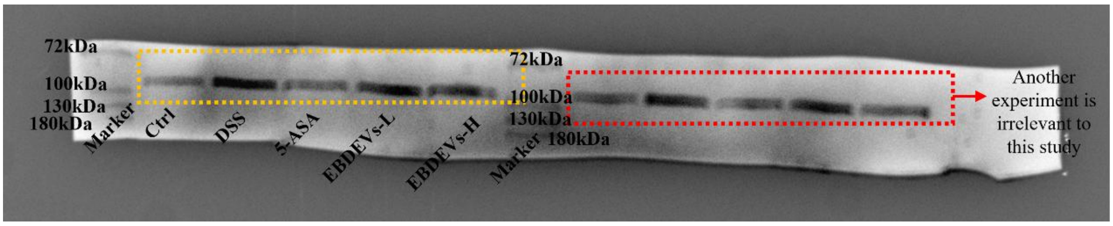

2. MyD88 (37kDa)

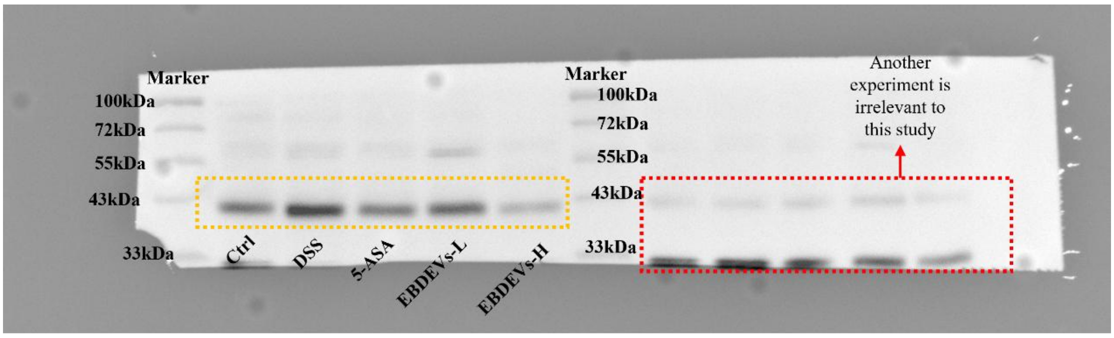

3. NF-κB p65 (65kDa)

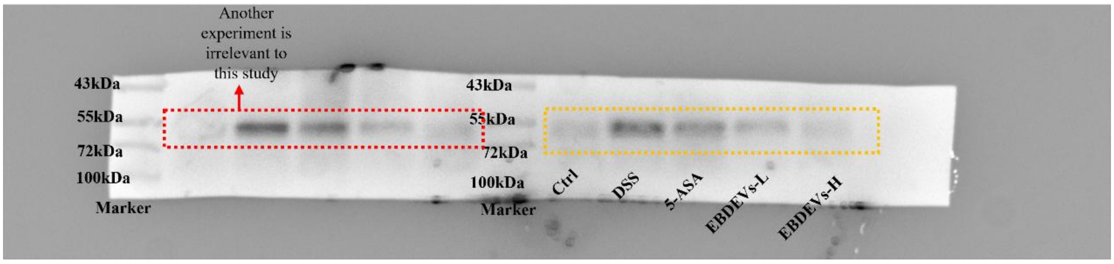

4. β-actin (42kDa)

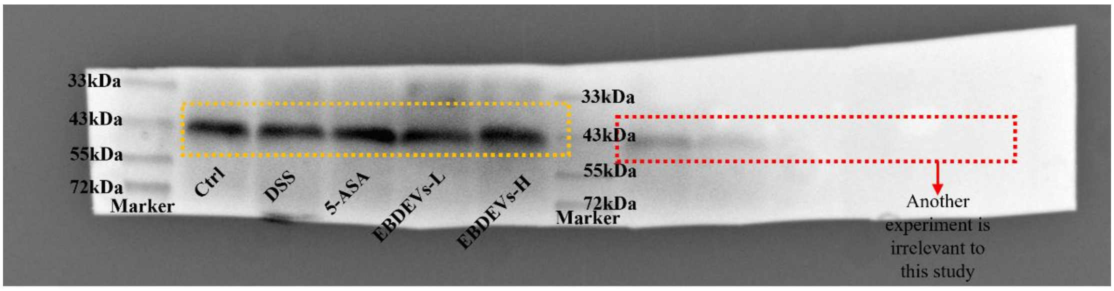

5. COX-2 (74kDa)

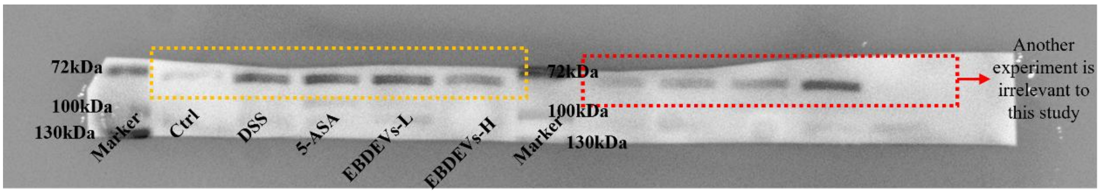

6. NLRP3 (118kDa)

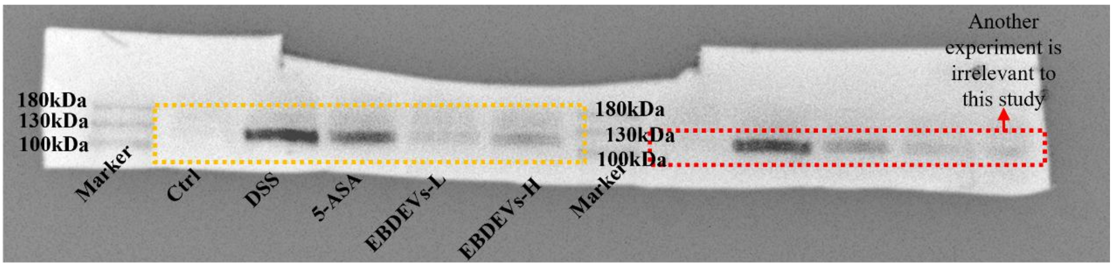

7. Caspase-1 (45kDa)

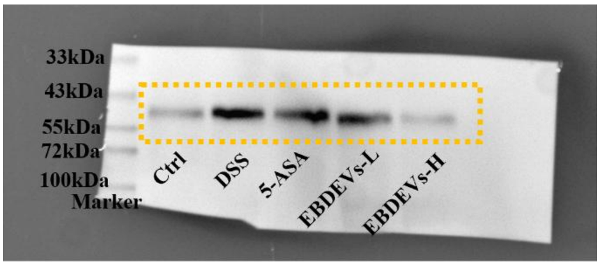

8. β-actin (42kDa)

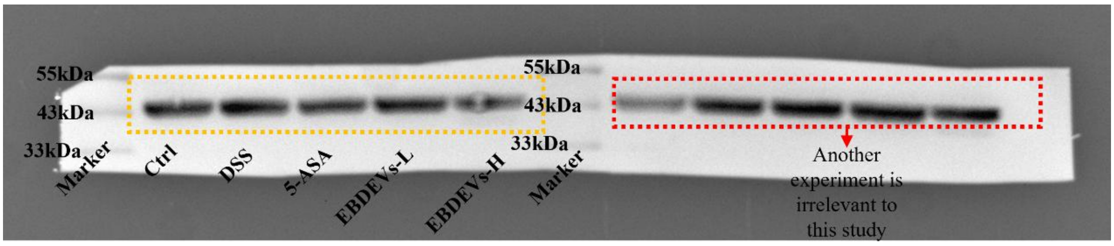

Supplement: Supplementary file 1 [file nanomaterials-16-00968-s001.zip › Supplementary Material S1.pdf]
